# Modeling the prediction of business intelligence system effectiveness

**DOI:** 10.1186/s40064-016-2525-6

**Published:** 2016-06-16

**Authors:** Sung-Shun Weng, Ming-Hsien Yang, Tian-Lih Koo, Pei-I Hsiao

**Affiliations:** Department of Information and Finance Management, National Taipei University of Technology, No.1, Sec. 3, Zhongxiao E. Rd., Taipei, 10608 Taiwan; Department of Information Management, Fu Jen Catholic University, No. 510 Zhongzheng Rd, Xinzhuang Dist., New Taipei City, 24205 Taiwan; Department of Accounting, Shih Chien University, No.70 Ta-Chih Street, Chung-Shan District, Taipei, 10462 Taiwan

**Keywords:** Business intelligence, Prediction model, Decision tree algorithm, Logistic regression analysis

## Abstract

Although business intelligence (BI) technologies are continually evolving, the capability to apply BI technologies has become an indispensable resource for enterprises running in today’s complex, uncertain and dynamic business environment. This study performed pioneering work by constructing models and rules for the prediction of business intelligence system effectiveness (BISE) in relation to the implementation of BI solutions. For enterprises, effectively managing critical attributes that determine BISE to develop prediction models with a set of rules for self-evaluation of the effectiveness of BI solutions is necessary to improve BI implementation and ensure its success. The main study findings identified the critical prediction indicators of BISE that are important to forecasting BI performance and highlighted five classification and prediction rules of BISE derived from decision tree structures, as well as a refined regression prediction model with four critical prediction indicators constructed by logistic regression analysis that can enable enterprises to improve BISE while effectively managing BI solution implementation and catering to academics to whom theory is important.

## Research motive and purpose

Business intelligence (BI) is an emerging topic (Jafar 2010; LaValle et al. 2011; Presthus and Bygstad 2012), and is the top priority on the agenda related to technology initiatives that covers 36 industries in 41 countries in 2012 (Gartner 2013). BI skills are extremely important to organizations (Brandel 2009). Successful enterprises leverage BI technology (Chaudhuri et al. 2011). However, empirical studies on BI remain scarce (Chen and Siau 2011; Jourdan et al. 2008). Therefore, one of the objectives of this study is to conduct a pioneering empirical investigation of a systematic method for the construction of rules for the prediction of business intelligence system effectiveness (BISE) in the context of BI implementation to forecast BI performance. The systematic work conducted in this study first determined measurement items of BISE, then adopted statistical analysis to create a prototype model of prediction and then conducted data mining techniques to form data structures and refined the prototype model to increase model predictive power.

Based on the prediction models with a set of rules for evaluation of the effectiveness of BI solutions, this study also attempts to help BI managers master the critical attributes of the BISE to achieve successful BI. From a BI solutions implementation perspective, the important issues facing an enterprise are to enhance BI capabilities via effectively monitoring BI solution implementation, including identifying critical indicators and assessing the BISE to measure BI performance and thus determine the direction of BI system improvement. Organizations require technical capabilities to achieve BI success (Işık et al. 2013). Although most BI systems integration, information delivery and analysis techniques have already been incorporated into the commercial BI and analytics platforms offered by Microsoft, IBM, Oracle etc. (Schlegel et al. 2013), the greatest challenge for most organizations is not technology, but rather the ability to apply or application of new technologies (McKenney et al. 1995). Previous studies have shown that investment in information technology has not yielded clear benefits in the context of transitional economies (Samoilenko 2008; Osei-Bryson and Ko 2004). Additionally, numerous academics and practitioners have evaluated the outcomes of BI implementation. Unsurprisingly, evaluation results regarding the contribution of BI to organizational performance have been inconsistent (e.g., Jourdan et al. 2008; Chaudhuri et al. 2011; Elbashir et al. 2013; Rubin and Rubin 2013; Brands 2014). While BI success remains unrealized in numerous organizations, this study sought to provide a direction to improve the implementation performance of BI solutions through effective management of the BISE. More importantly, from monitoring to mastering, the critical predictive indicators of the BISE are essential to BI success. If those indicators could be effectively managed by constructing prediction models and rules for assessing the BISE in the BI implementation, BI performance might improve. Therefore, this study attempts to resolve the above problems to help enterprises achieve BI success.

## Related theory

BI is a system of data conversion that ranges from immediate feedback to complex risk management (Wu et al. 2014). The main goal of BI implementation is to facilitate interactions between data management and information sharing to help analysts and managers with both analysis and task execution (Turban et al. 2010; Elbashir et al. 2013). Proactive BI includes real-time data warehousing, data mining, automated anomaly and exception detection, proactive alerting with automatic recipient determination, seamless follow-through workflow, automatic learning and refinement, geographic information systems and data visualization (Langseth and Vivatrat 2003). Consequently, BI systems combine data gathering, storage, access, analysis and knowledge management with analytical tools to present complex internal and competitive information for planners and decision-makers (Watson 2009; Yeoh and Koronios 2010), and thus help them make better and faster decisions (Mikroyannidis and Theodoulidis 2010; Chaudhuri et al. 2011). Furthermore, BI may support effective decision-making under time pressure and supplies accurate and useful information to appropriate decision-makers (Larson 2009), such as faster access to information, easier information query and analysis, higher interactivity and improved data consistency (Popovič et al. 2009). Information solutions providers such as Microsoft (2014) and IBM Cognos (2014) developed powerful BI tools and solutions to help enterprises increase the effectiveness of BI.

To measure system effectiveness, BI supports organizational decision-making in increasingly complex operating environments (Rubin and Rubin 2013), thus yielding multiple benefits in relation to BI implementation. BI comprises both technical and organizational elements that present its users with historical information for analysis to enable effective decision-making and management support (Işık et al. 2013). BI measurements generally serve two main purposes (Lönnqvist and Pirttimäki 2006): the first is to prove that BI solutions are worth the investment; the second is to help manage the BI process, namely to ensure the BI solutions satisfy user needs and that the process is efficient (Hou 2012). Four measurable benefits of BI: it can help avoid unnecessary costs, decisions based on good BI may increase revenues, BI information may help improve resource allocation decisions and thus maximize profitable investments and the direct link between a BI decision and business performance could be measured (Sawka 2000).

Additionally, the implementation of BI systems makes an organization more agile (Chen and Siau 2011). BI solutions enable enterprises understand their internal and external environments through systematic information acquisition, collation, analysis, interpretation and exploitation (Chung et al. 2005). Investing in BI solutions for marketing activities can help enterprises quickly launch products or services, satisfy consumer needs in relation to tracking systems, manage trade and customer interactions (Gessner and Volonino 2005; Watson and Wixom 2007), and help enhance marketing and sales (Vukšić et al. 2013). BI is used to understand firm capabilities, as well as the current status and future trends in the markets, technologies and regulatory environments relevant to a firm (Negash 2004).

To summarize, researchers and practitioners have argued that BI implementation has diverse effects. BI success represents the attainment of multiple benefits, such as improved profitability, reduced costs and improved efficiency (Işık et al. 2013). BI capabilities can help an organization improve both agility in the face of change and overall performance (Watson and Wixom 2007; Brands 2014). Therefore, for practical BI management reasons, to identify the critical elements of BISE and establish a predictive model and rules for the effectiveness of BI system implementation is extremely helpful.

## Research process

**Fig. 1:**
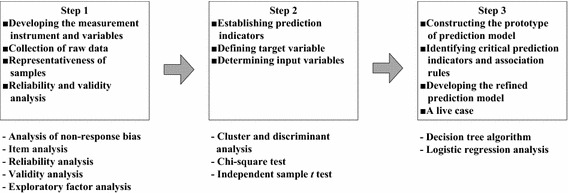
Research process

Three steps of the systematic research process were designed to construct the prediction rules and models of BISE. Figure 1 outlines the research process. First, this study developed a measurement instrument for data collection and conducted validity and reliability analysis to verify its accuracy and the internal consistency of the measurement items. Second, predictive indicators of BISE were determined. Finally, this study identified the critical prediction attributes and indicators and constructed prediction models and rules of BISE using data mining techniques and multivariate statistical methods. Logistic regression analysis and decision tree algorithm are two typical useful methods of data classification and forecasting (e.g., Schumacher et al. 2010; IBM 2016). The research results will provide practitioners a set of self-evaluation guidelines for BISE estimation.

The research focused on the Taiwanese financial services industry. Owing to the global financial services industry being impacted by the Basel II Accord and Sarbanes-Qxley Act, further promotion is needed of the application of new information technologies. For example, banks should comply with financial regulations, thus increasing demand for analytical tools such as intelligence systems and performance management systems.

## Methods and results

### Step 1

#### Developing the measurement instrument and variables

The measurement instrument comprised two parts: BISE and enterprise characteristics. Table 1 lists the variables of the measurement instrument.

**Table 1:** Research variables of BISE and characteristics of enterprise

Variables and descriptions	
***BISE***	
*Technological attributes*	
X_*1*_	Promotion of data availability
X_*2*_	Promotion of the heterogeneous information integration
X_*3*_	Promotion of the multi-dimensional data storage and display
X_*4*_	Promotion of ease of use of decision support systems
X_*5*_	Promotion of the rapid aggregation and expansion of information
X_*6*_	Promotion of information timeliness and flexibility
X_*7*_	Promotion of information applicability
*Human competencies attributes*	
X_*8*_	Promotion of the rapid decision-making
X_*9*_	Promotion of the ability to discover hidden problems
X_*10*_	Promotion of the rapid communication and monitor of exception information
X_*11*_	Promotion of the immediate responses of key performance indicators
X_*12*_	Promotion of the accumulation of business intelligence
X_*13*_	Promotion of the efficiency of decision support system maintenance
*Supports specific business processes attributes*	
X_*14*_	Support for service profitability
X_*15*_	Support for new service development and customer acquisition
X_*16*_	Support for service continuity and customer retention

Enterprise characteristics	
Industry categories	Banks, insurance companies, bills finance corporations, securities firms, trust business companies and investment companies
Company capital	Under 3 billion dollars (excluding), 3–10 billion dollars (excluding), 10–30 billion dollars (excluding), over 30 billion dollars
Number of years the business has been established	Under 10 years (excluding), 10–20 years (excluding), 20–30 years (excluding), over 30 years
Total number of employees	Under 300, 301–1000, 1001–5000, over 5001
Number of employees in information department	Under 10, 11–30, 31–100, over 101
Number of years of enterprise having implemented BI solutions	Under 1 year (excluding), 1–3 years (excluding), 3–5 years (excluding), 5–7 years (excluding), over 7 years

Previous studies on the evaluation attributes of BI benefits were diverse. The study defined BISE as performance when implementing BI solutions, including the effectiveness with which enterprises use information to improve and optimize business processes and decision support systems to derive multiple benefits of BI. The measurement items of BISE were based on viewpoints including the arguments of Watson et al. (2004) and Işık et al. (2013) that BI comprises both technical and organizational elements and that of Laursen and Thorlund (2010) who noted that the BI system has three elements: a technological element that collects, stores and analyzes data, as well as delivering information; a human competencies element dealing with human abilities to retrieve data and generate reports, generate knowledge and make decisions; and a third element that supports specific business processes for increasing business values. The work of developing measurement items comprised two steps: (1) integrating the attributes of BISE with related studies [e.g., Gessner and Volonino (2005); Microsoft (2007); Bitech (2007) etc.]; (2) based on the results of step 1, interviewing and discussing with two senior BI managers in the information divisions of Taiwanese banks to determine final measurement attributes and items of BISE. Table 1 lists three measurement attributes and 16 measurement items as research variables. Responses to each variable were assessed on a 5-point Likert scale, ranging from 1, indicating “strongly disagree”, to 5, indicating “strongly agree.”

Table 1 also lists the characteristics of enterprises surveyed in this study on whether or not the surveyed characteristics influence BISE. Enterprise characteristics were developed based on the perspectives of Rogers (2003) and Laudon and Laudon (2012), and the research variables included industry category, company capital, number of years the business has been established, total number of employees, number of employees in the information department and number of years of enterprise having implemented BI solutions.

#### Collection of raw data

A representative sample of 294 participants in the Taiwan financial services industry, including banks, insurance companies, bills finance corporations, securities firms, trust business companies and investment companies, all listed in the Taiwan Business Directory, were selected as research participants. Respondents were general managers or executives in information divisions. Before questionnaire distribution, two chief information officers reviewed the wording of questionnaire items. One questionnaire was distributed to each firm by mail or e-mail. A total of 294 questionnaires were distributed and 77 valid questionnaires were returned for a valid return rate of 26.2 %.

Respondents were followed up after 3 weeks to increase the response rate. This study also analyzed non-response bias. Responses were divided into two groups, the initial response group and the follow-up response group. The Chi square test was applied to both groups to identify differences in the six variables representing enterprise characteristics—industry category, company capital, number of years the business has been established, total number of employees, number of employees in the information department and number of years of the enterprise having implemented BI solutions. The test *p*-values were >0.05 at 0.172–0.995, indicating no significant difference between these two data groups.

#### Representativeness of samples

For the six industry characteristics, banks, insurance companies, bills finance corporations, security firms, trust business companies and investment companies, respectively, the numbers of population were 73, 51, 136, 13, 3 and 18; and the numbers of the returned valid samples were 23 (31.5 %), 13 (25.5 %), 25 (18.4 %), 4 (30.8 %), 3 (100.0 %) and 9 (50.0 %). This study further adopted Chi square test to examine the representativeness of the samples. The test *p* value was >0.05 at 0.106, indicating that no significant differences existed between these two groups (population and the returned valid samples), and existed sufficient sample sizes to achieve adequate representation.

The sample profile was such that participants were predominantly from the banking 29.9 % (n = 23) and securities industries 32.5 % (n = 25). Roughly 24.7 % (n = 19) of companies had capital reserves of NT$10–30 billion. Firms with 101–300 employees accounted for 28.6 % (n = 22) of the sample. Approximately 58.4 % of firms (n = 45) had 1–30 employees in their information departments, and 41.6 % (n = 32) had been established for 10–20 years. Finally, 23.4 % (n = 18) of firms had implemented BI solutions for 1–3 years; while 57.2 % (n = 44) had done so for >3 years.

#### Reliability and validity analysis

Item analysis identified measurement items that deserved to be retained versus those that needed to be revised or discarded. This study applied the *t*-test to two extreme groups, namely the highest and lowest scoring groups, using the internal consistency criterion. All measurement items of BISE had *p*-values of 0.000 (<0.05), indicating adequate discrimination and clarity.

The Cronbach’s α value for BISE was 0.955, and did not increase even after item exclusion. The item-to-total correlation of measurement items was in the range 0.650–0.831 (see Table 2), and the criterion of 0.35 was seen as an acceptable corrected item-total correlation, indicating the scale exhibited satisfactory reliability.

**Table 2:** Results of reliability analysis and exploratory factor analysis

Variables code	Cronbach’s α value if item deleted	Item-total correlation	Communality	Factor loading	Eigenvalue	Variance explained	Cronbach’s α value
*Factor 1*					9.637	60.234 %	0.950
X_*5*_	0.952	0.760	0.759	0.842			
X_*10*_	0.952	0.752	0.729	0.819			
X_*4*_	0.952	0.753	0.723	0.814			
X_*3*_	0.952	0.776	0.693	0.755			
X_*7*_	0.952	0.736	0.649	0.747			
X_*1*_	0.952	0.746	0.658	0.744			
X_*12*_	0.951	0.795	0.705	0.736			
X_*8*_	0.951	0.778	0.687	0.735			
X_*11*_	0.950	0.831	0.745	0.726			
X_*2*_	0.952	0.751	0.637	0.701			
*Factor 2*					1.438	8.990 %	0.903
X_*16*_	0.954	0.678	0.753	0.830			
X_*14*_	0.953	0.712	0.773	0.829			
X_*15*_	0.953	0.683	0.754	0.828			
X_*13*_	0.954	0.650	0.606	0.715			
X_*9*_	0.953	0.692	0.593	0.648			
X_*6*_	0.952	0.736	0.613	0.585			

Factor 1: the business information management effectiveness of BI

Factor 2: the decision support effectiveness of BI

Validity is the ability of a scale to measure what it is intended to measure, and to measure its accuracy in identifying key content characteristics. This study adopted exploratory factor analysis to assess the scale of BISE to verify its suitability for this investigation. Table 2 lists the analytical results. The Bartlett’s test of sphericity and the Kaiser–Mayer-Olkin (KMO) measure of sampling adequacy were first conducted to confirm the appropriateness of the sample data for factor analysis. Kaiser (1974) indicated that a KMO in the range 0.80–0.89 is meritorious. Since the factors were obtained through principle component analysis, the standards for factor selection were an eigenvalue exceeding one and a factor loading larger than 0.5 following varimax rotation. Analytical results for BISE demonstrated that the KMO was 0.892, and the Bartlett’s test of sphericity had a *p* value of 0.000 (<0.05). The cumulative variance explained was 69.224 %. Two factors were extracted, namely the business information management effectiveness of BI and the decision support effectiveness of BI. All 16 measurement items with communality were in the range 0.593–0.773 (>0.5) and factor loadings were in the range 0.585–0.842 (>0.5), indicating satisfactory construct validity.

### Step 2

#### Establishing prediction indicators

For constructing predictive models and association rules, the target variable and the input variables were used in the predictive analysis techniques should be determined and defined.

#### Defining target variable

To determine the target variable of the decision tree (DT) algorithm and logistic regression (LR) analysis, cluster analysis was performed on the sample of 77 respondents using two factors extracted by the exploratory factor analysis, namely the business information management effectiveness of BI and the decision support effectiveness of BI. The K-means method of non-hierarchical clustering analysis was adopted to classify the BISE of financial services firms in Taiwan. This study further applied discriminant analysis to validate the analytical results of cluster analysis, and found that they agree with the K-means method, and the cluster analysis had accuracy of 100.00 %.

Table 3 lists the results of cluster analysis for group one, comprising 26 firms, and group two, comprising 51 firms, performed using two factors of BISE, and conducts the *t*-test to demonstrate the significant difference in the two factors between the two groups. The analytical results showed that the *t*-values were 7.111 and 11.787, while the *p*-value was 0.000 (<0.05) and hence significant. The means of the two factors in group one were lower than in group two, indicating that group one had lower BISE and so was named the “low BISE group”, while group two had higher BISE and so was termed the “high BISE group,” and as the target variable of DT algorithm and LR analysis.

**Table 3:** Results of cluster analysis

Groups	Firms	Factor 1		Factor 2		Discriminant analysis to validate (%)
		*t*-value	*p*-value	*t*-value	*p*-value	
Group 1: low BISE group	26	7.111	0.000	11.787	0.000	100
Group 2: high BISE group	51					100

Factor 1: the business information management effectiveness of BI

Factor 2: the decision support effectiveness of BI

#### Determining input variables

Overfitting refers to the phenomenon whereby the numerous input variables of the DT algorithm and LR analysis make it easy to select unrelated variable categories. This study conducted the Chi square test and independent sample *t*-test to select meaningful input variables of statistics as the input variables of the DT algorithm and LR analysis to avoid deviation of the analysis results.

A Chi square test measures whether significant difference exists between the effects of six independent variables indicating enterprise characteristics on the target variable. Analytical results showed that the *p*-value (0.016) of the variable of number of years of enterprise having implemented BI solutions was below 0.05, indicating a significant correlation with the target variable. Meanwhile, the *p*-values of the remaining five variables exceeded 0.05 (range, 0.143–0.127), and the χ^2^ values were in the range 2.224–5.425, indicating no significant correlation with the target variable. Thus, only the variable of number of years of enterprise having implemented BI solutions was selected as the input variable of the DT algorithm.

Additionally, this study performed independent sample *t*-test on the target variable through 16 measurement items of BISE. The analytical results demonstrated that the *p*-values of 16 variables were all below 0.05, and the *t*-values were in the range 4.254–8.010, achieving significance, and thus these variables were adequate as input variables of the DT algorithm and LR analysis.

### Step 3

#### Constructing the prototype of prediction model

The LR analysis was used to model dichotomous outcome variables and forecast relationships between the dependent variable and a set of independent explanatory variables. The LR model constructed a two-way classification system as a substitute for linear discriminant analysis, and avoided the unreasonable assumption that the binary type covariance matrix must be equal (Reichert et al. 1983). Based on the all 17 input variables of the BISE and enterprise characteristics derived from the measurement instrument of step 2, this step first adopted LR analysis to model the influence and explanatory power of prediction variables.

Analytical results demonstrated that the value of Cox-Snell *R*^2^ was 0.583 and that of Nagelkerke *R*^2^ was 0.808, suggesting that the model had receivable prediction power. Table 5 also indicates that the four predictive performance measures, accuracy, precision, recall, and F1-measure, of the prototype LR model, were 89.61, 78.13, 96.15 and 86.21 %, respectively, leaving the effectiveness of the prototype unproven. Additionally, the two prediction variables were X_*15*_ and X_*13*_, with the estimated values of 3.703 and 3.408, and *p*-values were 0.003 and 0.006 (<0.05), respectively, which indicated good explanatory power. Meanwhile, under the prototype model, the remaining 15 prediction variables had less explanatory power. To improve the prototype model, this study first extracted the critical input variables and structured the association rules, and then constructed the refined LR model to enhance the predictive power and accuracy. The associated calculation work is presented below.

#### Identifying the critical prediction indicators and association rules

The DT algorithm was one of the methods used in data mining for knowledge discovery, and systematically analyzed the data to identify rules and relations for use in data classification and prediction (Han and Kamber 2006). This algorithm comprised a supervised learning method for data mining (Patterson 1990). The classification tree was adopted, and included parameter setting and standards for calculating divergence. Model accuracy was assessed using the actual DT performance to calculate the proportion correctly classified as judgment. This study administered the CART algorithms, and the splitting criteria, impurity measures and Gini criterion Breiman et al. (1984) were as described below (IBM 2016).

At node *t*, the optimal split *s* was selected to maximize a splitting criterion *Δi*(*s*,*t*). When the impurity measure (*i*(*t*)) for a node was defined, the splitting criterion corresponded to a decrease in impurity. *ΔI*(*s*,*t*) = *p*(*t*)*Δi*(*s*,*t*) was labeled the improvement.

$$i(t) = \sum\limits_{i,j} {C\left( {i\left| j \right.} \right)p} \left( {i\left| t \right.} \right)p\left( {j\left| t \right.} \right)$$

$$\Updelta i\left( {s,t} \right) = i\left( t \right) - p_{L} i\left( {t_{L} } \right) - p_{R} i\left( {t_{R} } \right)$$

*p*(*t*) The probability of a case in node *t*.* p*(*j*|*t*) The probability of a case in class *j* given that it falls into node *t*.* C*(*i*|*j*) The cost of miss-classifying a class *j* case as a class *I* case. *C*(*j*|*j*) = 0

**Fig. 2:**
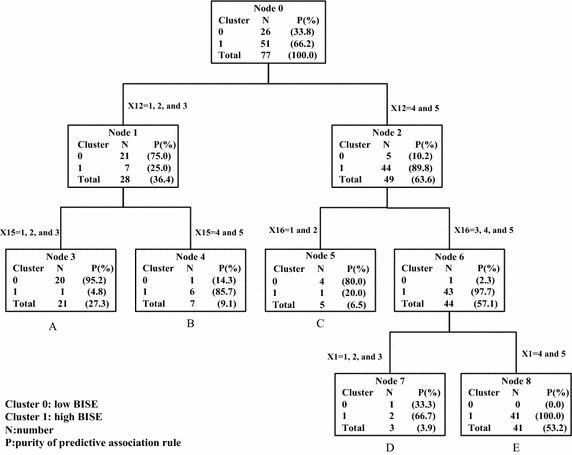
Results of tree structure of the DT algorithm

Analytical results demonstrated that the structure of DT that obtained the most accurate classification, including the minimum number of cases, was two in the total branching nodes and the maximum DT depth was five hierarchies. The performance measures, accuracy rate, precision rate, recall rate, and F1-measure rate, of the structure of DT were 94.81, 92.31, 92.31 and 92.31 %, respectively (see Table 5). Furthermore, Fig. 2 illustrates the tree structure of the DT algorithm. The details of each terminal node included description of rule paths, categories of belonging, numbers entering the node, and analysis of category purity. The tree structure was such that five paths (namely, association rules) existed from the root node to the leaf nodes.

- Rule A: If X_*12*_ = {1,2,3} and X_*15*_ = {1,2,3}, then low BISE. P = 95.20 %.
- Rule B: If X_*12*_ = {1,2,3} and X_*15*_ = {4,5}, then high BISE. P = 85.70 %.
- Rule C: If X_*12*_ = {4,5} and X_*16*_ = {1,2}, then low BISE. P = 80.00 %.
- Rule D: If X_*12*_ = {4,5}, X_*16*_ = {3,4,5} and X_*1*_ = {1,2,3}, then high BISE. P = 66.70 %.
- Rule E: If X_*12*_ = {4,5}, X_*16*_ = {3,4,5} and X_*1*_ = {4,5}, then high BISE. P = 100 %.

X*i* = variable of BISE

The extent of promotion of or support for X*i*; very low = 1, low = 2, medium = 3, high = 4, very high = 5

P = purity of predictive association rule

Analyzing all five prediction rules revealed that the first critical determining attribute and indicators of BISE is the variable X_*12*_: promotion of the accumulation of business intelligence is a human competency attribute. Based on rules A and B, the second is variable X_*15*_: support for new service development and customer acquisition. Meanwhile, rule C showed that the third is X_*16*_: support for service continuity and customer retention. The above two critical determining indicators are embedded in support for specific business processes attributes. Finally, the last is X_*1*_: promotion of data availability is a technological attribute, based on analysis of rules D and E.

#### Developing the refined prediction model

To improve the prototype prediction model constructed based on the first LR analysis in “Constructing the prototype of prediction model” section, this study adopted four critical prediction indicators (variables) of BISE derived from the classification and prediction model by using the DT algorithm to refine the prototype prediction model to enhance improve its predictive power and accuracy.

Analytical results demonstrated that the suggested prediction equation was as follows:

$$p = \frac{{e^{f(x)} }}{{1 + e^{f(x)} }} \,$$

$$\ln \left( {\frac{p}{1 - p}} \right) = f(x) = - 3 8. 4 8 2 { } + 2. 8 0 0 * X 1 2 { } + 2. 7 5 9 * X 1 5 { } + 3.862 * X 1 6 { } + 2.477 * X 1 { }$$

The probability (*P*) in the range 0–1 is used to identify the BISE in the BI implementation, where the value of *P* is close to 1 means high BISE and the value of *P* is close to 0 means low BISE. The four critical prediction indicators included X_*1*_, X_*12*_, X_*15*_ and X_*16*_, and achieved the good explanatory power (see Table 4). The variable of X_*16*_: support for service continuity and customer retention had the highest predictive power.

Based on the Hosmer–Lemeshow goodness of fit test, the χ2 value was 6.777 and the *p*-value was 0.561(>0.05), demonstrating that the model and data were suitable and the model had good overall fitness. The value of Cox-Snell *R*^2^ was 0.621 and that of Nagelkerke *R*^2^ was 0.860, which signified the LR model had good predictive and explanatory capability. The omnibus test χ2 was 74.641 and the *p*-value was 0.000(<0.05), which means the model was capable of predicting the BISE.

**Table 4:** Results of LR analysis

Variables	Estimate	Standard error	Wald χ^2^	*p*-values
Constant	−38.482	11.501	11.196	0.001***
X_*16*_: support for service continuity and customer retention	3.862	1.617	5.704	0.017**
X_*12*_: promotion of the accumulation of business intelligence	2.800	1.158	5.850	0.016**
X_*15*_: support for new service development and customer acquisition	2.759	1.208	5.219	0.022**
X_*1*_: promotion of data availability	2.477	1.120	4.892	0.027**
Model fit properties	Omnibus test χ^2^ = 74.864, *p*-value = 0.000***			
	Hosmer–Lemeshow test χ^2^ = 6.777, *p*-value = 0.561			
	Cox-Snell *R* ^2^=0.622			
	Nagelkerke *R* ^2^=0.862			

*** *p* < 0.01; ** *p* < 0.05

Additionally, Table 5 list the results of the four predictive performance measures—accuracy, precision, recall, and F1-measure of the refined LR model, and the comparison between actual conditions and test results of the two LR models and the structure of DT. For the refined LR model, the total predictive accuracy, precision, recall, and F1-measure were 94.81, 92.31, 92.31 and 92.31 %, respectively. The results demonstrated that the refined LR model exhibited a better predictive performance in terms of accuracy, precision, recall and F1-measure, than the prototype LR model, and the same predictive performance as the structure of DT. These two models had the same four predictive performance measures because of the small sample sizes; when the analyzed samples small, the probability that the two models are equally accurate is high.

**Table 5:** Results of predictive performance measures of the LR model and the DT structure

Methods	Groups	Actual condition	
		Low BISE group	High BISE group
LR model (prototype)	Test result		
	Low BISE group	25(TP)	7(FP)
	High BISE group	1(FN)	44(TN)
	Accuracy	89.61 %	
	Precision	78.13 %	
	Recall	96.15 %	
	F1-measure	86.21 %	
LR model (refined)	Test result		
	Low BISE group	24(TP)	2(FP)
	High BISE group	2(FN)	49(TN)
	Accuracy	94.81 %	
	Precision	92.31 %	
	Recall	92.31 %	
	F1-measure	92.31 %	
DT structure	Test result		
	Low BISE group	24(TP)	2(FP)
	High BISE group	2(FN)	49(TN)
	Accuracy	94.81 %	
	Precision	92.31 %	
	Recall	92.31 %	
	F1-measure	92.31 %	

*TP* true positive; *FP* false positive; *FN* false negative; *TN* true negative

#### A live case

##### Introduction

AC Company, a financial services firm, was founded in Taiwan in the 1990s. The company mostly provides loans and sale intermediation in the used equipment market. Recently, the company developed and implemented BI systems based on information technology (IT), substantially improving its business agility and operational innovativeness.

##### BI system architecture

The information system of AC Company includes customer relationship management systems, enterprise resource planning systems (loan management information systems and equipment financing information systems), supply chain management systems, internal auditing systems, administrative information systems, and financial management systems. The company uses data mining techniques, multidimensional database techniques, data warehouse tools, and SQL2008 tools to integrate all of these information systems into its BI systems.

The BI systems of AC Company are IT-driven and function-oriented systems for analyzing data and presenting actionable information to executives and decision-makers to help them make business and management decisions. BI systems include several data processing tools, and support operation and management applications that enable the company effectively to collect data from internal systems that support loan activities and operations, and external sources that are associated with suppliers, customers, government, and related organizations. BI systems also analyze or virtualize obtained data to create reports for executives and decision-makers that provide the results of analyses on which they can act.

##### BI system effectiveness

The implementation of BI systems has enabled AC Company to transform collected data into useful information and reports that help to improve decision-making and management, and to construct a featured knowledge database that accumulates BI and increases BISE. For example, recently, to cope with the competition in Chinese loan markets, the company adopted a business strategy to develop innovative loan service systems to improve customer service, and to use the aforementioned information systems and BI systems in its subsidiary in mainland China to open up new loan markets.

Executives of the company examined the effectiveness of implemented BI systems using the four key indicators of BISE, which were extracted by logistic regression analysis as follows.

- BI systems strongly support (level 4 on the Likert scale) service continuity and customer retention (X_*16*_).
- BI systems provide very effectively (level 5 on the Likert scale) promote the accumulation of business intelligence (X_*12*_).
- BI systems strongly support (level 4 on the Likert scale) new service development and customer acquisition *(*X_*15*_).
- BI systems provide very effectively (level 5 on the Likert scale) promote data availability (X_*1*_).

## Conclusions and suggestions

### Research conclusions

This study created two models for predicting BISE, including the DT structure and the LR equation. These two models provided different prediction rules for forecasting enterprise BISE. Additionally, the prediction models and rules identified four critical prediction indicators embedded in three different attributes of BISE, including one technological attribute—promotion of data availability; one human competencies attribute—promotion of the accumulation of business intelligence; two supporting specific business processes attributes—support for new service development and customer acquisition and support for service continuity and customer retention in the BI solutions implementation context.

The results of this study further revealed that the most important prediction indicator of BISE was the extent to which service continuity was adequately supported via the implementation of BI solutions. The better BI solution support for service continuity and customer retention, the better BISE is. If enterprise evaluation suggested that the implementation of BI solutions significantly enhanced their ability to ensure service continuity, BI performance is identified as high BISE. Promoting the accumulation of business intelligence via BI solution implementation is the second important prediction indicator, BISE would be improved if enterprises rapidly accumulate business knowledge and information via BI solution implementation. The third important prediction indicator of BISE was the support for new service development and customer acquisition via BI solution implementation. The more BI solutions support new service development, the higher the quality of BISE. Enterprises should integrate marketing activities and decisions into BI systems to quickly analyze opportunities and grab them with appropriate timing to develop new services or products. The quality of new services or products development can increase the effectiveness of obtaining new customers (Gessner and Volonino 2005). Additionally, promoting data availability via the implementation of BI solutions is another crucial prediction indicator. Enterprises should seek to increase the ability of executives to rapidly access accurate and credible information and data by implementing BI solutions to promote decision quality.

### Theoretical implications

Three theoretical implications are described below. First, this study adopted a systematic research process and quantitative techniques to model the prediction of BISE in order to overcome inadequacies in the empirical studies on BI, a neglected area in academic research (Jourdan et al. 2008; Chen and Siau 2011). Particularly, the authors are unaware of any empirical study focused on modeling the prediction of BISE. Using the systematic research process and methodology, this study proposed critical attributes and indicators for predicting and assessing the effectiveness of BI implementation. This study thus complemented the gap in the research on the BI implementation context.

Second, in terms of information technology adoption theory, this study provided an answer to the question of how to effectively construct and acquire the critical indicators of BISE. The main results of this study also provided a sequential research opportunity to study the BI performance in depth. Applying the prediction models and rules of BISE as an empirical research bridge can explore the future influence of BI solution implementation on, for example, organizational performance and innovation performance. The prediction model of BISE can be further expanded using influences on BI performance to establish a comprehensive research model for new BI solution implementation.

Finally, the systematic research process demonstrated that two different predictive models and rules were constructed using data mining techniques and statistical methods that could provide high predictive accuracy in BISE. The DT algorithm identified important attributes of measurement that could be used to refine the prototype of the LR model to improve the predictive accuracy of model.

### Practical implications

The results of this study have practical implications for enterprises seeking to effectively manage critical attributes and indicators that determine BISE. BI project managers can borrow the prediction models and rules to guide their assessment in the development of self-evaluating tools of BISE to improve BI system performance and boost BI success. Based on the predictive results of BISE, mangers can quickly develop improvement strategies for BI solution implementation to effectively enhance enterprise BI capabilities and competitiveness through high performance in BI system functions and services, and to complement existing management information systems that add value to system services and satisfy more complex decision-making support demands. Recognizing these key indicators and attributes of BISE should also help enterprises effectively utilize limited resources to improve BI performance and assist enterprises to develop differentiated BI functions for sustainable BI performance to acquire competitive advantage.

Additionally, when enterprises implement BI systems, they should consider their own business characteristics to develop a complete constructive plan based on the prediction models and rules of BISE, to design a suitable framework and content of BI systems, invest in sufficient resources, and effectively execute and supervise BI project to ensure BI success. The results of study can also help BI solutions providers to enhance quality of BI solutions. To use the prediction models and rules of BISE, BI solutions providers can focus on industry characteristics to expand the customerized BI solutions and improve counseling services to assist enterprises in effective resource allocation and strengthen BI performance to maximize decision-maker satisfaction and enhance BISE.

Moreover, from the resource-based perspective, Amit and Schoemaker (1993) argued that enterprise competitive advantage is based on the cultivation and exploitation of internal resources and abilities. A BI system is not a short-term management information system, but rather a long-time implementation to yield permanent performances and benefits. If enterprises implement BI solutions for longer, problems such as how to improve information processing and quality of system-decisions can be solved, and enterprise BI capabilities will be continuously enhanced.

## Limitations and suggestions for future research

Limitations of this study included the fact that the Taiwanese financial services industry is not a large industry, and thus contains a limited number of enterprises, including banks, insurance companies, bills finance corporations, securities firms, trust business companies, and investment companies. BI system applications and tools are not suitable for evaluating BISE. For example, Microsoft’s BI Tools, IBM BI solutions, SAP BI and Big Data mining tools, and Oracle BI solutions. All of these applications and tools focus on helping firms to process, analyze, and mine data and to report information, to improve their decision-making. Therefore, future research may address the development of simulation tools and applications of BISE for organizational BI system managers. Additionally, based on the innovation diffusion perspective of Kwon and Zmud (1987), development of information systems proceeds through six stages—initiation, adoption, adaption, acceptance, routinization and infusion. Consequentially, BI systems implementation undergoes several developmental stages. Future research on the construction of performance prediction models and rules for each life stage of BI systems can be conducted to help enterprises assess the outcomes of implementing BI systems as a foundation for improving BI system effectiveness. Finally, Premkumar et al. (2005) argued that environmental uncertainty, complexity and dynamics influenced demand for information processing and further influenced the adoption and implementation of new information technology for enterprises. Therefore, the impact of influences such as external factors on the effectiveness of BI solution implementation deserves further investigation.
